# Signal characteristic alterations of carotid artery dissection on high resolution magnetic resonance imaging: a follow-up study

**DOI:** 10.1186/1532-429X-13-S1-P393

**Published:** 2011-02-02

**Authors:** Maryam Etesami, Ye Qiao, Robert J Wityk, Bruce A Wasserman

**Affiliations:** 1 Russell H. Morgan Department of Radiology and Radiological Science, Johns Hopkins University, Baltimore, MD, USA; 2 Department of Neurology, Johns Hopkins University, Baltimore, MD, USA

## Introduction

Carotid artery dissection (CAD) is one of the most common causes of stroke in young and middle aged adults. Magnetic resonance imaging (MRI) is commonly used for evaluation of suspected CAD.

## Purpose

We sought to determine the over time changes in contrast enhanced MRI characteristics of CAD that have not been established before.

## Methods

Five patients (4 male, age range 33-55 years) with proven spontaneous CAD were scanned at baseline (within 2 weeks of symptom initiation) and at follow-up using high resolution MRI. Axial two dimensional ECG gated double inversion recovery T1 weighted (T1W) turbo spin echo images were acquired before and after administration of 0.1 mmol/kg gadolinium (acquired resolution: 0.35x0.35x2mm^3^). Degree of stenosis, presence intraluminal thrombosis, intramural hematoma (IMH) and perivascular enhancement (PVE) were evaluated on both scans. On matched images, regions of interest (ROI) were selected for each of the above mentioned components if present. Contrast enhancement of each component was calculated as percent difference of signal to noise ratios (i.e. signal intensity of the selected ROI divided by standard deviation of noise) on pre and post contrast T1W images.

## Results

Twenty three MR images in five patients were quantitatively analyzed for contrast enhancement. Mean interval between two scans was 7.8 weeks (Range 4-13 weeks) while all patients except one were receiving anticoagulation with Warfarin. Only one case had complete occlusion at baseline with an intraluminal thrombosis which was completely recanalized on the follow-up scan with minimal residual stenosis after 10 weeks. All cases showed improvement in the degree of stenosis at follow-up. IMH was detected as T1 hyperintensity within the thickened vessel wall in pre-contrast images in 4 cases, 3 of which were completely resolved on the second scan and one had small IMH residue. In contrast to IMHs that did not show any contrast enhancement (-0.5%± 5.7%), the corresponding vessel wall areas on follow-up scans demonstrated enhancement (35.1%± 4.3%) (p value<0.001). PVE was evident in all cases on both baseline scans (81.4%± 36.3%) and follow-up scans (76.8%± 19.4%) even in cases with complete disappearance of IMH and minimal residual narrowing. However, degree of PVE was slightly lower at follow-up compared to initial scan (p value>0.05).

## Conclusions

Non-enhancing intramural hematoma in CAD can resolve in a few weeks and corresponding vessel wall areas on follow-up scans demonstrated enhancement. Perivascular enhancement may remain longer on MRI.

